# Occurrence of PFAS in Cow’s Milk: A Comparative Study of Swedish Farms near Contaminated Sites and Regional Dairy Production Facilities

**DOI:** 10.1021/acs.jafc.5c07211

**Published:** 2026-02-27

**Authors:** Anders Glynn, Jennifer Nyström Kandola, Gunnar Johanson, Carolina Vogs, Carl Ekstrand, Maria A Karlsson, Hasitha Priyashantha, Karl Lilja, Lars-Erik Karlsson, Leo Wai-Yin Yeung, Anna Kärrman, Ida Hallberg

**Affiliations:** † Department of Animal Biosciences, 8095 Swedish University of Agricultural Sciences, Box 7054, Uppsala 750 07, Sweden; ‡ Institute of Environmental Medicine, Karolinska Institutet, Box 210, Stockholm S-171 77, Sweden; § 116272 The Federation of Swedish Farmers, Franzéngatan 1B, Stockholm 105 33, Sweden; ∥ Environmental Pollutants Unit, Swedish Environmental Protection Agency, Stockholm SE-106 48, Sweden; ⊥ 692206 Växa Sverige, Box 288, Uppsala SE-751 05, Sweden; # Man-Technology-Environment (MTM) Research Centre, School of Science and Technology, Örebro University, Fakultetsgatan 1, Örebro SE-701 82, Sweden; ∇ Department of Clinical Sciences, Swedish University of Agricultural Sciences, Uppsala SE-750 07, Sweden

**Keywords:** per- and polyfluoroalkyl substances, milk, dairy, cattle, bovine, food safety

## Abstract

Per- and polyfluoroalkyl substances (PFAS) are persistent pollutants that raise food safety concerns, especially near contamination hotspots. This study measured 9 PFAS in milk and 15 PFAS in water from 22 Swedish dairy farms <10 km from contamination hotspots and 49 PFAS in milk from 20 regional production facilities. PFOA, PFOS, and PFNA were quantified in 5–77% of milk from dairy farms, with maximum levels of 18, 17, and 10 pg/g milk ww, respectively; the remaining PFAS were below method detection limits (MDL). All PFAS were < MDL at production facilities. One dairy farm milk sample exceeded EU’s indicative level for PFOA (10 pg/g), but levels in production facilities suggest limited consumer exposure. No correlation was found between PFAS in farm water and milk, implying other exposure routes may dominate when water contamination is low. While results indicate limited health risks, contamination in other milk-producing regions cannot be ruled out, supporting the need for continued PFAS monitoring in dairy production.

## Graphical abstract

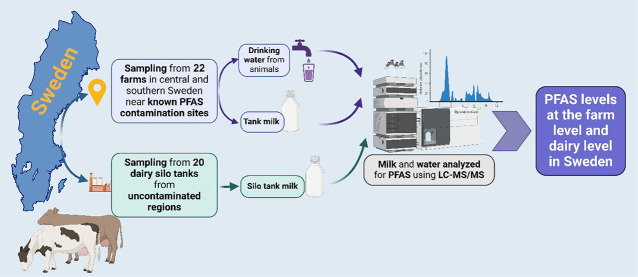

## Introduction

Some per- and polyfluoroalkyl substances (PFAS) are considered as persistent organic pollutants (POPs) under the Stockholm Convention. They have been detected all over the world in the environment, wildlife, and humans.^1^ In humans, detectable levels of PFAS in some cases are likely affecting human health.^2–4^ The global spread of PFAS pollution is related to atmospheric transport and deposition as well as global ocean circulation. Widespread use of PFAS-containing products and discharge from manufacturing to disposal have contributed to the ubiquitous pollution of the Earth.^5^

In Sweden, PFAS pollution is widespread, and several compounds have been detected in various abiotic and biotic matrices,^6,7^ in certain cases above the current Environmental Quality Standards (EQS) for PFOS in surface water (0.65 ng/L) and fish (9.1 μg/kg) set by the EU, which are based on earlier toxicological standards.^8^ Moreover, many local areas are highly polluted, especially by perfluoroalkyl acids (PFAAs) that are highly mobile and persistent in the environment.^2,9^ This pollution has become a major food and drinking water safety concern, and a fraction of children, adolescents, and adult populations in Europe have sums of their dietary intake of PFAS4, namely perfluorooctanoic acid (PFOA), perfluorononanoic acid (PFNA), perfluorohexanesulfonic acid (PFHxS), and perfluorooctanesulfonic acid (PFOS), that are exceeding the tolerable weekly intake (TWI) of 4.4 ng/kg bodyweight established by the European Food Safety Agency (EFSA).^10^

The European Commission established maximum levels for these four individual PFAAs and also the sum of the four PFAAs (PFAS4) in foods of animal origin, including meat, offal, eggs, and fish/shellfish (EU 2023/915).^11^ The maximum limit values for food products came into force on January 1, 2023. For milk, no limit values exist yet, but indicative levels for the PFAS4 have been issued.^12^ The indicative levels are 0.060 μg PFHxS/kg, 0.020 μg PFOS/kg, 0.050 μg PFNA/kg, and 0.010 μg PFOA/kg, corresponding to 60, 20, 50, and 10 pg/g, respectively.^12^ If the indicative levels in milk samples are exceeded, investigations into the causes of contamination should be carried out.^12^ In Sweden, the concentrations of perfluorohexanoic acid (PFHxA), perfluoroheptanoic acid (PFHpA), PFHxS, PFOA, PFOS, PFNA, perfluorodecanoic acid (PFDA), perfluoroundecanoic acid (PFUnDA), perfluorododecanoic acid (PFDoDA), perfluorotridecanoic acid (PFTrDA), and perfluorotetradecanoic acid (PFTeDA) in milk have generally been reported to be very low, i.e., below the EU indicative levels for PFAS4.^13^ In a study of PFAS levels in pooled milk samples from the food control program of the Swedish Food Agency (1999–2010), only PFOS was detected in the samples, at levels ranging from <3.5 pg PFOS/g ww milk to 7.3 pg PFOS/g ww milk,^13^ and thus below the indicative level for PFOS in milk (20 pg PFOS/g milk). However, certain areas in Sweden have been found to be highly contaminated with PFAS, mainly due to their close proximity to firefighting training facilities where PFAS-containing firefighting foams have historically been used.^9^ It may be suspected that some dairy farms have experienced PFAS contamination, with PFOA, PFNA, PFOS, or PFHxS levels above the indicative level, similar to findings from studies of milk from contaminated sites in China,^14^ Germany,^15^ and the USA.^16^ Given the widespread consumption of dairy products in Sweden and the potential overlap between PFAS-contaminated areas and dairy production sites, it is crucial to ensure that these types of foods are safe for consumption. Although several studies have reported PFAS in food products, few have investigated tank milk from individual farms in the vicinity of PFAS hotspots or compared these with large-scale storage tanks from regional processing facilities covering wide areas. This knowledge is essential to guide future prioritization of risk management efforts to address possible PFAS contamination in the food chain.

The aim of this study was therefore to screen milk samples from dairy farms located near PFAS-contaminated areas in Sweden and to compare the observed levels to the background PFAS concentrations in milk from regional dairy processing facilities across the country. We also compared the PFAS levels to the recently established EU indicative thresholds for PFOA, PFNA, PFOS, and PFHxS. Finally, the contribution of PFAS exposure from cattle drinking water was assessed to evaluate its role in milk contamination at the farm level.

## Materials and Methods

### Study Design

Dairy farms within a 10 km radius of known PFAS hotspots in central and southern Sweden were identified and invited to participate. At each of the 22 participating farms, samples were collected from on-site bulk milk storage tanks and the drinking water supply for the cattle. In addition, silo tank milk samples were collected from 20 dairy processing facilities geographically distributed across Sweden. These facilities receive pooled milk from multiple surrounding farms, making silo samples representative of regional milk production. All milk and water samples were analyzed for PFAS and compared to the EU indicative thresholds for PFOA, PFNA, PFOS, and PFHxS.

### Identification of PFAS Hotspots

Farms were included from regions with known PFAS contamination of groundwater and/or surface water, hereafter referred to as “PFAS hotspots”. There is no PFAS production industry in Sweden. Local contamination sites (PFAS hotspots) have been discovered following the use of PFAS-containing firefighting foam.^9,17^ PFAS hotspots were identified based on investigations initiated by the Swedish Armed Forces. Several locations in midsouthern Sweden were selected where elevated levels of PFAS had been detected in surface water, groundwater, and/or drinking water following the historic use of PFAS-containing firefighting foam. Figure S1 in the (Supporting Information SI) illustrates the approximate location of the PFAS hotspots in the midsouth parts of Sweden selected for the study, which cover six areas (A–F). ***Area A:*** Two military fire drill areas were identified where PFAS-containing firefighting foam was used during 1960–1990. In groundwater at one of these areas, elevated concentrations of PFAS (perfluoropentanoic acid (PFPeA), PFHxA, PFHpA, PFOA, perfluorobutanesulfonic acid (PFBS), and PFHxS) were detected, with a sum reaching 5,000. In surface water, the sum reached 340 ng/L. These levels were found during investigations carried out on behalf of the Swedish Air Force in 2015.^18^ ***Area B:*** Historic use of firefighting foam occurred at a fire drill area near the Armed Forces Technical School (FMTS). Elevated groundwater concentrations of the sum of PFBS, PFHxS, PFOS, 6:2-fluorotelomersulfonic acid (6:2 FTS), perfluorobutanoic acid (PFBA), PFPeA, PFHxA, PFHpA, PFOA, PFNA, and PFDA (PFAS11), up to 33,900 ng/L, were reported from sampling near the drill area between 2016 and 2018. The direction of groundwater distribution was estimated to primarily occur toward the south and west, where concentrations of PFAS11 ≥ 30 ng/L were detected along the full extension of a stream draining the training site.^19^ ***Area C:*** In autumn 2013, PFAS11 > 9,000 ng/L, dominated by PFOS and PFHxS, was found in outgoing drinking water originating from a water reservoir near a military airfield. The source of contamination was traced to a fire drill site, and the contamination plume reached the drinking water recipient in a north-to-southeast direction with PFOS concentrations up to 4,000 ng/L.^2^ ***Area D:*** The training site of the Swedish Air Force had a historic use of PFAS-containing firefighting foam. Groundwater within the area was sampled during 2014 and 2016, revealing high levels (with a maximum concentration of PFAS11 at 1,500,000 ng/L during 2016). The water samples outside the testing sites indicated a sporadic contamination pattern, with levels ranging from a few ng/L to >100,000 ng/L.^20^ ***Area E:*** Elevated concentrations of PFAS (maximum level of PFAS11: 98 ng/L)^21^ were detected in a drinking water plant in 2012 and traced to a fire drill area at an airport. After installation of a carbon filter, average levels of PFAS4 dropped to 13 ng/L and PFAS11 to 22 ng/L.^22^ ***Area F:*** A training area of the Swedish Armed Forces where PFAS-containing firefighting foam was likely used historically. Sampling at several locations within the training site during 2015 and 2016 revealed levels >9,000 ng PFAS11/L in groundwater and surface water (maximum level in surface water was 600,000 ng PFAS11/L). Close to the stream draining the area, a concentration of 11,000 ng PFAS11/L was observed.^23^

### Recruitment of Farms

Farms located within a 10 km radius in any direction from a PFAS hotspot were asked to participate in the study. The farmers received written and oral information about the project and signed a consent form if they agreed to participate. The distance between the farm and the PFAS hotspot was recorded; however, to maintain farm anonymity, the direction from the hotspot was not recorded. The farmers received a questionnaire regarding water sources, feed sources, and animal husbandry (available in the SI, Table S1). All farm identity data and personal data recorded were handled according to the General Data Protection Regulation (GDPR). The researchers processing the data could not connect the samples to a specific farm or a specific region in Sweden and only accessed the information on the distance between the sampled farm and the PFAS hotspot.

### Milk and Water Sampling at Farms

Bulk milk storage tank samples were collected from recruited farms (*n* = 22) in 50 mL HD-PE tubes in May–June 2023. The sample tube was rinsed with milk prior to collection. Tap water samples from the main cattle drinking water source were collected in 2L HD-PE bottles. Sampling was done after the tap had been running for 1 min, and the bottle was rinsed with water prior to sampling. At the majority of the farms (70%), some animal groups, such as heifers intended for on-farm replacement, could access other water sources during pasture (lake, sea, or stream). In 22% of the farms, lactating cows could also access other water sources during pasture. Field blanks for water and milk sampling were collected by keeping a sampling tube or bottle open in the same area during the sampling procedure. Milk and water samples were transported chilled or frozen to the laboratory and stored at −20 °C until chemical analysis. Detailed sampling instructions are available in the SI, Table S2.

### Milk Collection at Dairy Processing Facilities

Raw milk samples from 20 dairy production plants across Sweden were collected from well-filled silos containing untreated milk during October 2022. A few liters of milk were flushed to clear residual content. Samples were collected in polypropylene bottles previously tested for PFAS, either directly or via a stainless-steel container, depending on the facility access. Bottles were partially filled to allow for expansion, sealed tightly, and stored refrigerated. Samples were then packed and transported chilled for analysis.

### Analytical Methods

#### Chemicals and Reagents

Native and isotopically labeled PFAS standards included in the targeted analysis were purchased from Wellington Laboratories (Guelph, Canada). Initially, we targeted a total of 16 PFAS in water and milk from the dairy farms, including 11 perfluorocarboxylic acids (PFCAs: C4–C14), four perfluoroalkanesulfonic acids (PFSAs: C4, C6, C8, C10), and perfluorooctane sulfonamide (FOSA). Both linear and branched isomers of PFOS were included, and branched isomers were quantified when the signals were ten times the noise (S/N > 10) by using the linear PFOS isomer and averaging the concentrations from transitions *m*/*z* 498.97 > 79.96 and *m*/*z* 498.97 > 98.90. The number of final reported PFAS was 15 for water and nine for milk because of low recovery or interfering peaks. Results are provided in the QA/QC section.

Analytical reagent-grade ammonium hydroxide (NH_4_OH, 25%), HPLC- (for extraction), and LC-MS- (for instrumental analysis) grade methanol (MeOH, ≥99.8% and ≥99.9%, respectively) were obtained from Fisher Scientific (Ottawa, Canada). Solid-phase extraction (SPE) cartridges were weak anion exchange (WAX) cartridges (Oasis WAX, 60 mg, 3 mL, 30 μm) from Waters Corporation (Milford, USA). Graphitized carbon (ENVI-Carb) was purchased from Supelco, Sigma-Aldrich (St. Louis, USA), and LC-MS-grade ammonium acetate was also obtained from Sigma-Aldrich. Laboratory-produced ultrapure water (18.2 MΩ·cm) was used throughout the experimental procedures.

#### Preparation of Dairy Farm Samples (Water and Milk)

Solid-phase extraction (SPE) was employed for water samples using mixed-mode weak anion exchange sorbents (OASIS WAX, 150 mg, 6 mL, Waters Corporation) following ISO 21675 with some modifications.^24^ To 250 mL of each water sample was added 10 μL of internal standard (1 ng; see Table S3 for details), and the pH was adjusted to 4 using acetic acid. The WAX sorbents were conditioned sequentially with 4 mL of 0.1% ammonium hydroxide in methanol, 4 mL of methanol, and 4 mL of ultrapure water. Samples were then loaded onto the SPE cartridges, followed by a washing step using 10 mL of ultrapure water, 4 mL of 25 mM ammonium acetate solution (pH 4), and 4 mL of 20% methanol in ultrapure water. The cartridges were subsequently dried under vacuum for 1 h.

To minimize potential losses of PFAS due to adsorption to the container walls, each sample container was rinsed with 4 mL of 0.1% ammonium hydroxide in methanol. This rinsate was used to elute the analytes from the SPE cartridges. The eluate (4 mL of 0.1% ammonium hydroxide in methanol) was collected in a 15 mL polypropylene (PP) tube and evaporated under a gentle nitrogen stream to a final volume of less than 0.5 mL. An injection standard (10 μL containing 1 ng) was added to the extract, and the volume was adjusted to 0.5 mL. Half of the extract was transferred to an LC vial and further evaporated to 80 μL, followed by the addition of 120 μL of 2 mM ammonium acetate in ultrapure water. The injection standards are the corresponding mass-labeled standards used to check the recoveries of the mass-labeled internal standards (Table S3).

Milk samples were processed by an in-house validated method partly based on US-FDA C-010.03, using acid precipitation followed by SPE and an additional cleanup using graphitized carbon. Briefly, 2 g of each milk sample was weighed into a 15 mL PP tube, and 5 μL of an internal standard (1 ng) was added. Subsequently, 2 mL of formic acid (50% v/v in ultrapure water) was added. The mixture was vortexed and subjected to ultrasonic treatment for 15 min, followed by centrifugation at 5,000 rpm for 30 min. The supernatant was transferred to a new 15 mL PP tube and diluted with ultrapure water to a final volume of 15 mL.

The SPE-WAX procedure was then performed in the same manner as that for water samples, up to and including the vacuum drying step. For additional cleanup, a preconditioned Envi-Carb sorbent was connected inline with the WAX cartridge before elution. The analytes were eluted using 4 mL and an additional 1 mL of 0.1% ammonium hydroxide in methanol. The combined eluates were evaporated under a gentle nitrogen stream to a volume of less than 0.5 mL. The extract was transferred to an LC vial, 5 μL of injection standard (1 ng) was added, and the volume was further reduced to 200 μL. Finally, 120 μL of 2 mM ammonium acetate in ultrapure water was added to each vial.

#### Instrumental Analysis and Quantification of Farm Samples (Water and Milk)

PFAS were analyzed using ultraperformance liquid chromatography coupled to a tandem mass spectrometer (UPLC-MS/MS; Acquity XEVO TQ-S, Waters Corporation, Milford, USA). Chromatographic separation was performed on a BEH C18 column (100 × 2.1 mm, 1.7 μm; Waters Corporation) maintained at 50 °C. A column between the pump and injector was used to retain PFAS originating from the system (Waters Corporation PFC Isolator column). The mobile phase A was composed of 70% 2 mM ammonium acetate in water and 30% methanol, while mobile phase B consisted of 2 mM ammonium acetate in methanol. The flow rate was set to 0.3 mL/min, and the injection volume was 10 μL. Multiple reaction monitoring (MRM) mode was used to enhance selectivity, with at least two transitions monitored for each analyte where available (Table S3). Exceptions were PFBA and PFPeA, for which only one transition could be monitored. Detailed information on the gradient program and MS/MS parameters can be found in Aro et al. (2021).^25^

#### Quality Control of Dairy Farm Samples (Water and Milk)

Accuracy and precision were assessed by spiking native PFAS compounds (1 ng) into water samples in triplicate (Table S4, SI). Milk analysis accuracy was evaluated for four PFAS by triplicate analysis of an in-house reference material^26^ used in an interlaboratory study^26^ and compared to the study’s consensus values (Table S4, SI). For each batch of samples, extraction blanks (consisting of ultrapure water) were included to monitor the contamination. No detectable level of PFAS in the method blank for water analysis was found, although several PFAS showed detected levels in the extraction blank for the milk samples; the levels ranged from a few picograms up to 1000 picograms for PFBS. Instrument blank injections were made after each set of 8–10 injections, and reinjection was made when a sample showed a signal higher than the highest concentration in the calibration curve to monitor possible carry-over. For most analytes, isotopically labeled internal standards were available and used for each water and milk sample; in the absence of a corresponding labeled analogue (e.g., for PFDS and PFTrDA), alternative mass-labeled standards were applied. The recovery of internal standards was evaluated for all analytes (Table S4), excluding PFBS, PFDS, PFDoDA, PFTrDA, and FOSA, for which no corresponding mass-labeled injection standards were available.

In milk, the recovery of the internal standards ranged from 64% to 124%, and in water, it ranged from 63% to 98% (Table S4, SI). Method detection limits (MDLs) were calculated from extraction blanks and defined as the average concentration of the blank samples plus three times the standard deviation of replicate blank measurements. When no PFAS was detected in the blanks, the MDL was estimated from the lowest calibration point (LOQ), defined as the signal-to-noise ratio >10, after accounting for the concentration factor (Table S4, SI). Reported PFAS concentrations are not blank-corrected. Signals below the calculated MDL were quantified only for PFOS, PFHxS, PFNA, and PFOS and reported as estimates to, despite their uncertainty, introduce less statistical bias in comparative analyses.^27^

#### Analysis of Dairy Production Facility Samples (Milk)

A total of 49 PFAS (Table S6), including PFOA, PFNA, PFHxS, and PFOS, were analyzed in silo milk samples from regional production facilities. The chemical analysis was conducted by the accredited commercial laboratory Eurofins Food & Feed Testing Sweden AB (Lidköping, Sweden). As the analysis was performed externally, the method description provided here reflects the key validated steps documented by Eurofins rather than the full in-house protocol. Sample preparation followed the QuEChERS extraction method as previously described.^28^ Prior to weighing, samples were heated to 38 °C for 20 min to ensure homogeneity. Two grams of milk were used for extraction. Cleanup was performed exclusively with dispersive SPE. The extract was subsequently concentrated to 300 μL before instrumental analysis.

Identification and quantification were carried out using LC-MS/MS. Method parameters, including chromatographic separation, ionization mode, and MRM transitions, were optimized according to Eurofins validated internal protocols for food matrices. Quantification relied on isotopically labeled internal standards. For analytes lacking a specific labeled analogue, structurally similar or coeluting labeled compounds were employed as surrogate ISTDs to correct for matrix effects and instrument variability.

Native spike recoveries in the matrix for the reported PFAS generally ranged from 80% to 120%. A single sample was spiked at a concentration of 0.1 ng/g to assess recovery at the reporting level, while only one spiked replicate was performed in this study; the results were consistent with Eurofins’s validation data for milk. Method detection limits were set at one-third of the limit of quantification (LOQ), based on peak shape and measurement uncertainty. The reporting limit corresponds with the LOQ (Table S6). To ensure analytical integrity, both the reagent and instrument blanks were included in each analytical batch to monitor contamination.

### Statistical Methods

Statistical analyses were carried out in R (ver. 4.4.0; R Development Core Team). All statistical significance tests used a level of *p* ≤ 0.05. Relations between concentrations in milk, concentrations in water, and distances to hotspots were assessed with Spearman‘s Rank Correlation Test and linear regression. Lastly, we estimated the contribution of PFAA from exposure sources other than water, given the concentration found in water and the transfer of PFOA, PFNA, and PFOS from water to milk. To this end, we used biotransfer factors of 0.0112, 0.0155, and 0.0214 days/kg for PFOA, PFNA, and PFOS, respectively (eq 1, calculated from Vestergren et al., 2013).^29^

1 $$BTF\left[\frac{day}{kg}\right]=\frac{\mathrm{Concentration in food product}\;\left[ng\;PFAS/kg\right]}{\mathrm{total intake}\;\left[ng\;PFAS/day\right]}$$

## Results and Discussion

### Farms

The farms represented typical Swedish dairy farms. A total of 22 farms participated, comprising both organic (*n* = 5) and conventional (*n* = 17) production systems. The average herd size was 104 lactating cows, with a range from 17 to 280 cows. Herds included various breeds, although Swedish Red and Swedish Holstein were the most common. The average energy-corrected milk (ECM) yield was approximately 11,000 kg per cow per year, aligning with national averages.^30^ Most farms produced their own forage, and any supplementary feed was primarily sourced and purchased locally.

### Screening of Milk Coming off Farms near PFAS Hotspots

The results from the screening of PFAS in milk samples from dairy farms within 10 km of a local PFAS hotspot and from milk storage tanks at dairy production facilities are presented in Figure 1 and Tables S5–S6. Of the nine PFAS congeners reported in the 22 dairy farm milk samples, only PFOA, PFNA, PFHxS, and PFOS had detectable and quantifiable concentrations (Figure 1). The highest concentration found was for PFOA at ∼18 pg/g ww, followed by PFOS and PFNA at ∼17 and 10 pg/g ww, respectively (Figure 1; Table S5). PFHxS concentrations were below the MDL (8.11 pg/g) but still estimated in milk, with concentrations ranging between approximately 2 and 8 pg/g ww.

**Figure 1:**
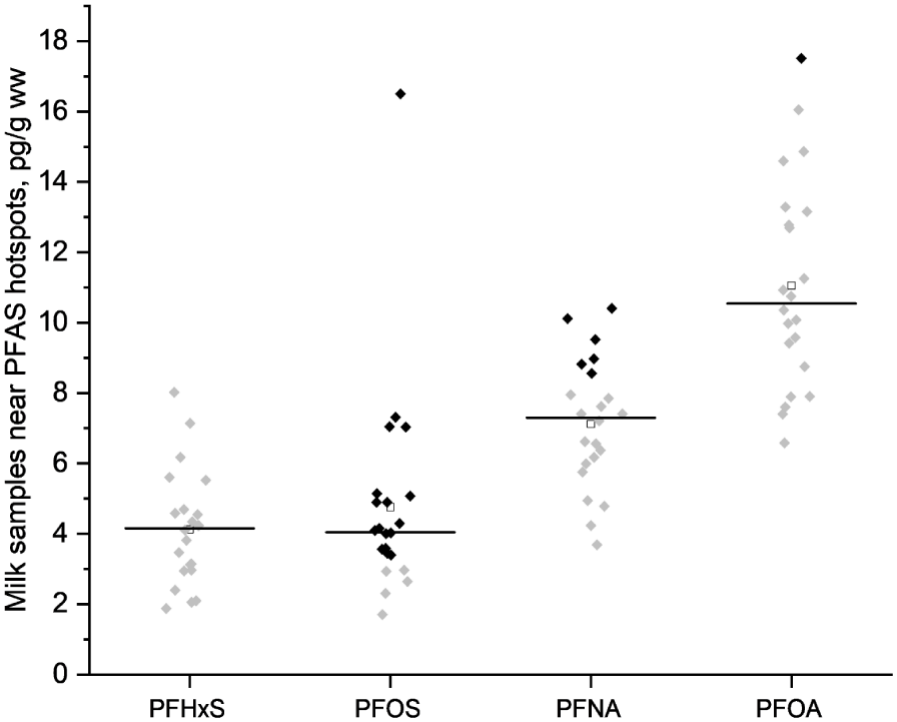
PFAS4 in milk samples from 22 dairy farms situated <10 km from PFAS hotspots. Black horizontal line represents medians, black squares represent values above the method detection limit (>MDL), and gray squares represent quantified values below the MDL. Quantified values are not blank-corrected.

In a previous Swedish study of two analytical batches of pooled samples from farm milk transport tanks, each with pooled milk from several farms sampled between 1999 and 2010, PFOS concentrations were similar (medians 4 to <4.4 pg PFOS/g) to the present study (median 4 pg PFOS/g) (Table S7).^13^ In the same study, PFNA could not be quantified in the transport tank milk with concentrations <7.3 pg PFNA/g milk, whereas 6 dairy farms in the present study had slightly higher quantifiable concentrations above 8.1 pg PFNA/g milk (median < MDL, range < MDL-10.4 pg/g). Comparisons of PFOA and PFHxS concentrations in the 1999–2010 transport tanks (MDL = 6.1 pg/g for PFOA and 2.3 pg/g for PFHxS) and the current study are hampered by the higher MDL in the present study (MDL: 16.7 for PFOA and 8.1 for PFHxS) (Table S4). The results nevertheless suggest that the PFAS4 concentrations in milk from storage tanks of farms close to PFAS hotspots were within the range of background concentrations. Thus, the proximity to PFAS hotspots did not seem to markedly influence the levels of PFAS in milk. This is supported by PFAS4 concentrations reported in a small study of retail milk samples (Table S7).^31^ Our screening presented here was limited to farms in proximity to only a few of the hundreds to thousands of confirmed or suspected PFAS hotspots in Sweden and does not exclude the possibility that there are some farms producing milk highly contaminated with PFAS in Sweden.^32^ One dairy farm in our study exceeded the EU indicative level of 10 pg of PFOA/g of milk; the farm measured the highest concentrations of both PFOA (17.5 pg/g) and PFOS (16.5 pg/g) in milk (Table S5). The highest PFOA level is an indication for further investigation of causes of the contamination according to the EU legislation. Our screening project was, however, not an official food-control project, and lack of funding hindered us from further investigating the cause of PFOA contamination besides field blank and laboratory blank controls, of which none confirmed contamination from sampling or analysis. PFOA has a short half-life in lactating cattle and is not associated with previous AFFF use, but a few previous studies have reported higher PFOA levels than those in our study (Table S7). Hypothetically, PFOS found in the milk may be associated with previous AFFF use, which was the basis for its inclusion in the study.

A few studies outside Sweden have screened for PFAS in milk from farm tanks or from individual cows (Table S7).^14,15,33–37^ Most studies report low median or mean PFAS4 concentrations similar to those in our study (Table S7). However, some studies reported substantially higher concentrations (Table S7). For instance, recent studies from Germany and China reported maximum concentrations of individual PFAS4 average values as high as 40–500 pg/g (Table S7).^15,33^ The German studies included one survey of 9 milk farms in a PFAS-contaminated area^15^ and another survey of 219 German farms with no information about potential PFAS contamination near the farms (Table S7).^15^ The Chinese study sampled individual cows and bulk storage tanks from eight farms in one area of Xinjiang in China.^33^ The results from both Germany and China suggest that some milk-producing farms have been affected by local PFAS contamination, leading to PFAS4 concentrations far above the EU indicative level. Given the scope of this study, contamination of other milk-producing areas cannot be ruled out and supports continued PFAS monitoring in dairy production also in Sweden.

### Screening of Drinking Water from Dairy Farms near PFAS Hotspots

The results of the screening for PFAS in water samples are shown in Figure 2, and details can be found in Table S8, SI. PFOA, PFNA, PFHxS, and PFOS were detected in all water from all farms, PFBS in six farms, and PFHxA in two farms. PFPeA and PFHpA were detected only in one of the farms. The highest concentration of PFAS4 (5.2 ng/L) was found at a farm that also had clearly elevated concentrations of PFHxA (1.0 ng/L) and PFBS (2.8 ng/L). PFAS4 levels were low on the other 21 farms (mean: 0.19, range: 0.006–1.85 ng/L). The widespread use of PFAS has contributed to contamination beyond major hotspots, leading to a low-level contamination (<10 ng/L) of drinking water worldwide.^2,38^ It therefore appears that the farms’ drinking water was not markedly affected by the local PFAS hotspot. Recruitment of farms did not consider the direction of the groundwater plume from the known PFAS hotspots, and it seems that contaminated water originating from the hotspot did not reach the wells of the selected farms. Consequently, a good understanding of water catchment areas and groundwater movements is needed to better understand to what extent PFAS hotspots may contaminate cattle drinking water and milk.

**Figure 2:**
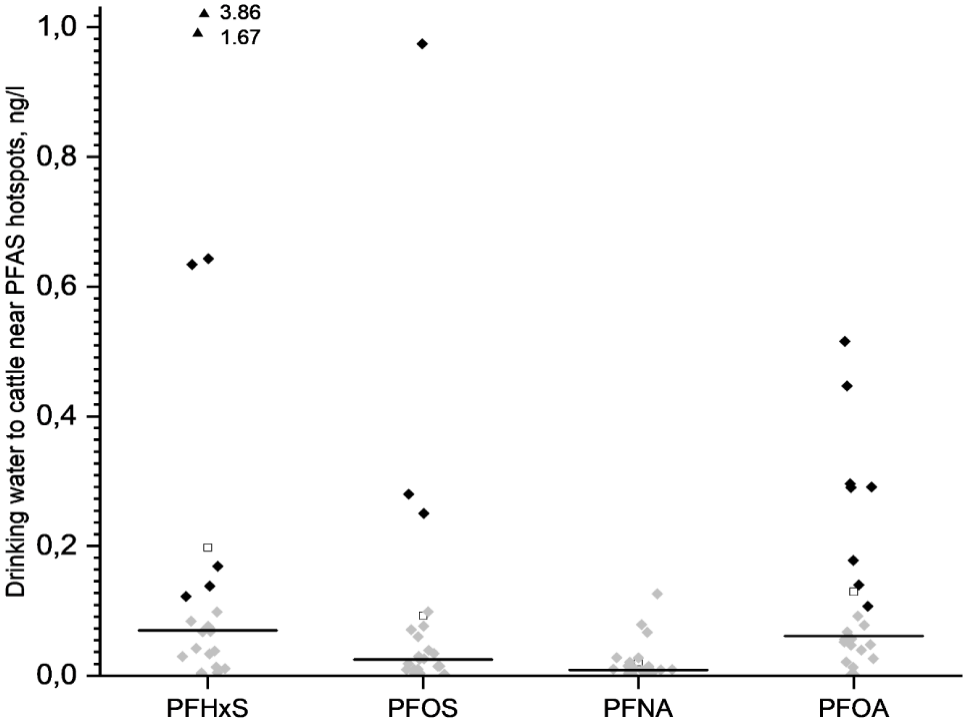
PFAS4 in cattle drinking water in farms <10 km from PFAS hotspots. The black horizontal line represents the median, each black square represents values above the method detection limit (>MDL), and gray squares represent quantified values below MDL. Extreme values above the axis range are depicted with a triangle and value.

### Dairy Production Facilities Samples

Of the 49 PFAS, including PFOA, PFNA, PFHxS, and PFOS, none could be quantified in the milk from the 20 dairy production facilities. The screening of these dairy production facilities (Table S6) revealed that PFAS4 concentrations were all below the EU indicative levels (PFOA: 10 pg/g; PFNA: 50 pg/g; PFHxS: 60 pg/g; and PFOS: 20 pg/g),^12^ demonstrating that these 20 dairy production facilities were not markedly influenced by PFAS contamination, although contributions from individual farms may have been diluted in the pooled milk.

Considerably more studies have screened for PFAS in retail milk samples than in dairy milk storage tanks (Table S7).^31,33,37,39–45^ It is important to bear in mind that contamination of milk might occur at all stages, from the PFAS accumulation in the cow to the possible PFAS contamination during storage, processing, and packaging, making comparisons between individual cow milk, farm tank milk, and retail milk difficult. Comparisons are further complicated due to a large variability in the level of detection and quantification between studies (Table S7). Nevertheless, the concentrations observed in our present study fall within the range of average values previously reported (Table S7). A possible exception is a study from Poland, which reported PFAS levels approximately ten times higher than those in our study, despite the absence of known contamination sources.^42^ However, a more recent investigation involving individual cows from five distinct regions in Poland did not observe such elevated concentrations.^35^

Several farm-level studies have reported individual milk samples with PFAS concentrations exceeding the EU indicative levels; however, such exceedances have not been documented at the dairy level. This discrepancy is likely attributable to the pooling of milk from multiple farms during processing, thereby diluting contaminated milk from one or a few farms with noncontaminated milk from many more farms.

### Factors Associated with PFAS in Milk

High-producing dairy cows can drink over 100 L of water daily, and the association between PFAS in drinking water and PFAS in milk was therefore investigated. Spearman’s Rank (Table 1, individual farm data in Tables S5–S6) and linear regression analyses (Figure 3) revealed no correlation between any of the four PFAAs substances in drinking water and milk. Moreover, using the biotransfer factor to estimate the concentration of PFAS in milk based on concentrations in drinking water suggested that the contribution from the sampled drinking water was insignificant compared to that from other sources (Table S9, SI). The contribution from other sources to PFOA, PFNA, and PFOS in milk exceeded 99% in nearly all 22 farms, except PFOA on 5 farms (96.5–98.7%), PFNA in one farm (98.9%) and PFOS in three farms (88.8–98.4%). Not surprisingly, the farm with the lowest contribution of PFOS from other sources (farm no. 14, 88.8%) was also the one with the highest PFAS4 level in water (5.2 ng/L). Our findings suggest that PFAS in the main cattle drinking water source did not contribute substantially to the PFAS levels in milk. Therefore, information on other exposure sources not accounted for in our study is needed to explain the variation in PFAS concentrations in milk. For example, at some of the farms, the lactating cows could access additional drinking water sources (stream, sea, lake) during pasture. Feed may also be an important source. Vestergren and colleagues (2013) estimated that the intake of PFOA, PFNA, and PFOS was highest via silage, barley, and supplements, and lowest via water.^29^ Contamination of cattle feed can occur in multiple ways. For example, accidental application of highly contaminated fertilizers on cropland intended for production of animal feed resulted in high concentrations of PFAS in hay and silage.^46^ Pastures or grass from contaminated floodplains have also been reported to have elevated PFAS concentrations.^47^ Atmospheric deposition could potentially also be a route of contamination of agricultural soil, where PFAS uptake in cattle can occur either directly from accidental consumption of soil via grass foraging or via uptake from crops intended as feed.^48^

**Table 1:** Spearman’s Rank Correlation Analysis of PFAA in 22 Dairy Farm Milk Samples and Water Samples in Dairy Farms across Sweden (*N* = 22).^a^

	ρρ	*p*-value
PFOA	–0.16	0.48
PFNA	0.01	0.95
PFHxS	0.35	0.11
PFOS	–0.21	0.33

a PFAA samples below the detection limit were also included in the analysis.

**Figure 3:**
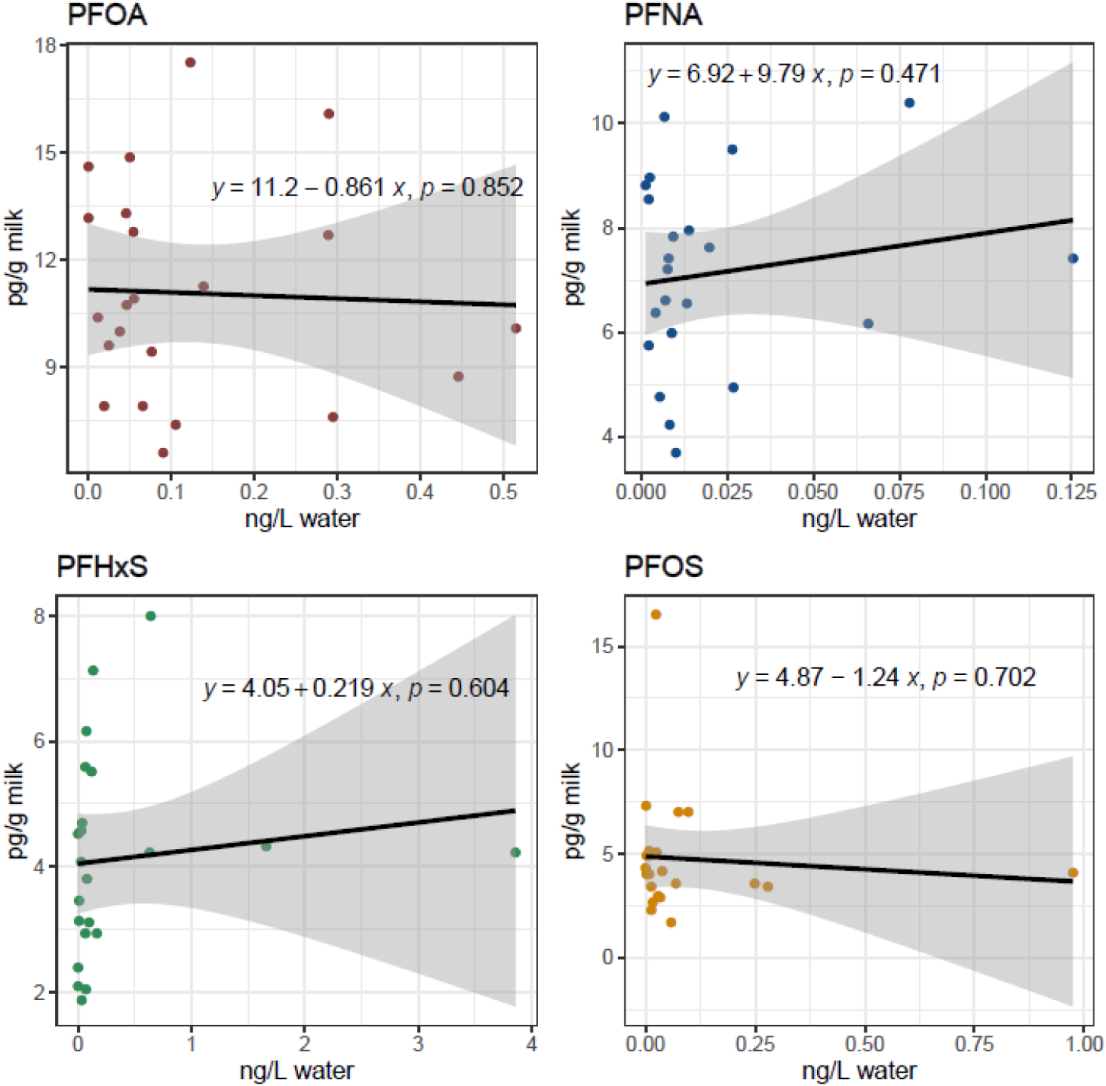
Linear regression analyses of PFOA, PFNA, PFHxS, and PFOS in milk (pg/g ww) and water (ng/L). Dots represent individual samples, black solid lines represent best fits, and gray bands 95% confidence intervals.

Spearman’s Rank and linear regression did not reveal any correlation between the farms’ distances from identified contamination sites using Spearman (Table S10), but it should be pointed out that farms were randomly recruited in all directions around each hotspot. Recruitment along the groundwater plumes (if they had been known) would have increased the chances of establishing a correlation.

## Conclusion

In conclusion, analyses of milk and drinking water from 22 dairy farms near known PFAS-contaminated sites in Sweden, along with 20 dairy silo samples, indicated generally no quantifiable or very low PFAS levels in Swedish milk. Although milk from one individual farm exceeded the EU’s indicative level for PFOA, PFAS was not detected in milk silo samples from dairy processing facilities, which represent pooled milk intended for retail and thus more accurately reflect actual consumer exposure. The low levels of PFAS in drinking water, together with the lack of correlation between PFAS concentrations in drinking water and milk from the same farms, indicate that other exposure pathways are likely more relevant for cattle PFAS intake on the participating farms and further support a limited impact from the nearby contamination hotspot. This limited screening near PFAS hotspots does not rule out the possibility of more severe PFAS contamination on other farms. Furthermore, the low indicative value for compounds like PFOA may fall below typical MDLs, LODs, and LOQs, complicating accurate detection and risk assessment.

## Supplementary Material



## Notes

Approximate location of the samples collected (Figure S1); the questionnaire (Table S1); sampling instructions (Table S2); analyzed compounds (Table S3); comparison of repeatability (RSD) and recovery in PFAS analyses (Table S4); PFAS in milk samples from 22 dairy farms (Table S5); PFAS in milk samples from 20 production facilities (Table S6); published literature (Table S7); PFAS in drinking water to lactating cows (Table S8); estimated PFAS concentrations in milk based on PFAS concentrations in cattle drinking water (Table S9); and correlation between milk PFAS concentrations and distance to hotspots (Table S10) (XLSX)

## Abbreviations and Nomenclature

LOD: limit of detection
LOQ: limit of quantification
MDL: method detection limit
MRM: multiple reaction monitoring
PFAAs: perfluoroalkyl acids
PFAS: per- and polyfluoroalkyl substances
PFBS: perfluorobutanesulfonic acid
PFHxS: perfluorohexanesulfonic acid
PFOS: perfluorooctanesulfonic acid
PFBA: perfluorobutanoic acid
PFPeA: perfluoropentanoic acid
PFHxA: perfluorohexanoic acid
PFHpA: perfluoroheptanoic acid
PFOA: perfluorooctanoic acid
PFNA: perfluorononanoic acid
PFDA: perfluorodecanoic acid
6:2 FTS: 6:2-fluorotelomersulfonic acid
PFAS11: sum of PFBS, PFHxS, PFOS, 6:2 FTS, PFBA, PFPeA, PFHxA, PFHpA, PFOA, PFNA, and PFDA
POPs: persistent organic pollutants
PP: polypropylene
QC: quality control
SPE: solid-phase extraction
TWI: tolerable weekly intake
UPLC-MS/MS: ultraperformance liquid chromatography coupled to a tandem mass spectrometer
